# CaReS-BiNet: A multi-scale deep learning framework for ECG arrhythmia classification

**DOI:** 10.1038/s41598-026-58810-6

**Published:** 2026-06-23

**Authors:** Manisha, Karunakar A. Kotegar, Shavantrevva Bilakeri

**Affiliations:** 1 School of Computer Science and Engineering, REVA University, Bengaluru, Karnataka 560064 India; 2 Department of Computer Applications, Manipal University Jaipur, Jaipur, Rajasthan 303007 India; 3 Manipal Institute of Technology, Manipal Academy of Higher Education, Manipal, India

**Keywords:** Cardiology, Computational biology and bioinformatics, Engineering, Health care, Mathematics and computing, Medical research

## Abstract

Automated electrocardiogram (ECG) arrhythmia classification remains challenging due to morphological complexity, severe class imbalance, and poor model generalization across heterogeneous datasets acquired under varying clinical conditions. To address these challenges, this paper proposes CaReS-BiNet, a Convolutional and Residual Squeeze-and-Excitation Bidirectional-LSTM Network that integrates parallel multi-scale one-dimensional convolutional branches, residual connections, lightweight Squeeze-and-Excitation channel attention, and bidirectional LSTM temporal modelling into a unified end-to-end framework. This design enables joint learning of local morphological features and long-range temporal dependencies directly from ECG heartbeat segments, with Gaussian noise injection employed to improve robustness to class imbalance and signal variability. Evaluated on the MIT-BIH Arrhythmia Database under the AAMI EC57 five-class protocol, CaReS-BiNet achieves an accuracy of 98.74%, precision of 98.70%, recall of 98.73%, and F1-score of 98.71%, outperforming the majority of compared state-of-the-art methods on recall and F1-score. Independent evaluation on the PTB Diagnostic ECG Database yields 99.31% accuracy, 99.27% precision, 99.16% F1-score, and an AUC of 0.999, demonstrating superior performance across all reported metrics against a recently proposed hybrid architecture evaluated on the same dataset. This robustness is further supported by consistent performance on an additional heterogeneous ECG dataset, achieving 98.08% accuracy. Binary ventricular ectopic beat (VEB) and supraventricular ectopic beat (SVEB) detection further confirms clinical reliability, with SVEB precision of 97.99% substantially exceeding existing methods. A systematic ablation study validates the individual contribution of each architectural component across both datasets. These results establish CaReS-BiNet as an effective framework for automated arrhythmia classification across diverse ECG datasets.

## Introduction

Cardiovascular diseases (CVDs) are the leading cause of death globally, accounting for an estimated 19.8 million deaths in 2022, which represents approximately 32% of all global deaths according to the World Health Organization (WHO) and related global health reports^1^. Of these deaths, more than four out of five are due to coronary heart disease and stroke, which often share common risk factors such as hypertension, tobacco use, unhealthy diet, physical inactivity, and harmful use of alcohol^2^. A significant proportion of premature deaths from CVDs occur in people under the age of 70, particularly in low- and middle-income countries, where access to early detection and effective preventive care is more limited. Cardiac arrhythmias, a key subset of cardiovascular diseases characterized by abnormal heart rhythms, contribute substantially to the global cardiovascular disease burden^1^. Their clinical significance arises from a strong association with life-threatening complications, including stroke, heart failure, and sudden cardiac death. Consequently, early and accurate detection of arrhythmias is essential for timely clinical intervention and improved patient outcomes.

Electrocardiography (ECG) is the most commonly used non-invasive diagnostic tool for monitoring cardiac electrical activity and identifying abnormal heart rhythms^2,3^. ECG signals provide valuable information about the timing and morphology of cardiac cycles, enabling clinicians to detect various types of arrhythmias. Owing to its low cost, ease of acquisition, and suitability for continuous monitoring, ECG has become indispensable in both clinical and ambulatory settings. However, the interpretation of ECG recordings is a complex and time-consuming task that requires extensive clinical expertise^4^. The increasing volume of ECG data generated through long-term monitoring and wearable devices further exacerbates this challenge, making manual analysis impractical and prone to inter-observer variability. Automated ECG analysis systems have therefore attracted considerable attention as a means of assisting clinicians in arrhythmia detection and diagnosis. Early approaches to automated ECG classification relied primarily on traditional signal processing techniques and machine learning algorithms^5,6^. These methods typically involve handcrafted feature extraction, such as time-domain, frequency-domain, or wavelet-based features, followed by conventional classifiers including support vector machines, k-nearest neighbors, decision trees, or shallow neural networks. For instance, Alvarado et al.^5^ employed time-based compression features for arrhythmia classification, demonstrating competitive ventricular ectopic beat detection but reporting substantially limited supraventricular ectopic beat precision of 56.68%, reflecting the fundamental difficulty of minority class detection with handcrafted feature pipelines. Similarly, Qurraie et al.^6^ combined handcrafted ECG features with a conventional classifier, achieving high overall accuracy but reporting considerably lower precision and F1-score, indicating that despite strong accuracy, the method struggles with balanced classification across minority arrhythmia classes. While such approaches have demonstrated promising results under controlled conditions, their performance is highly dependent on the quality of manually designed features and domain-specific knowledge. Moreover, handcrafted features often fail to fully capture the complex morphological variations and temporal dependencies inherent in ECG signals, limiting their robustness and generalization capability^7^.

Another major challenge in automated ECG classification is the inherent class imbalance present in arrhythmia datasets^8^. Clinically significant abnormal beats often occur far less frequently than normal rhythms, leading to skewed class distributions that can bias learning algorithms toward majority classes^9^. This imbalance is particularly problematic in real-world scenarios, where rare but critical arrhythmias must be detected reliably. Deep learning models that achieve high overall accuracy may still fail to generalize to minority classes if not designed carefully^10^. Several studies have proposed data augmentation and cost-sensitive learning to address these issues, highlighting that class imbalance can lead to over-fitting and poor minority class prediction if not properly mitigated^11^. Addressing class imbalance while maintaining robustness across heterogeneous datasets therefore remains a crucial research challenge.

To overcome these limitations, the advent of deep learning has significantly transformed the landscape of ECG signal analysis by enabling end-to-end learning directly from raw or minimally preprocessed signals^12^. Convolutional neural networks (CNNs), in particular, have been widely adopted due to their ability to automatically learn hierarchical feature representations and capture local morphological patterns such as QRS complexes, P waves, and T waves^10,13–15^. One-dimensional CNN architectures have proven effective in extracting discriminative features from ECG signals, leading to substantial improvements over traditional machine learning methods^13,15–20^. For instance, Oliveira et al.^18^ proposed a CNN-based architecture achieving 95.30% accuracy on the MIT-BIH database under the AAMI five-class protocol, yet reported precision of 50.04% and F1-score of 51.37%, highlighting that high overall accuracy can mask severe minority class misclassification and underscoring the insufficiency of accuracy as a sole evaluation metric under class-imbalanced conditions. Kiranyaz et al.^15^ introduced a patient-specific CNN framework that adapts model parameters to individual patient characteristics, achieving strong ventricular ectopic beat detection performance. However, this patient-specific paradigm requires retraining for each new individual, making it impractical for population-level deployment in real-world clinical settings where patient-specific training data may not be available at inference time. Nevertheless, despite their effectiveness in local feature extraction and morphological pattern learning, CNNs primarily focus on local spatial features and remain inherently limited in modeling long-range temporal dependencies, which are crucial for accurate arrhythmia classification.

To address this limitation, researchers have increasingly integrated recurrent neural networks (RNNs) with CNN-based architectures^21^. RNN variants such as long short-term memory (LSTM) and gated recurrent unit (GRU) networks are well suited for sequential data modeling and have demonstrated strong performance in capturing temporal dependencies in ECG signals^22^. Bidirectional extensions of these networks further enhance performance by exploiting contextual information from both past and future signal segments^23,24^. Hybrid CNN-RNN models have thus emerged as a dominant paradigm in ECG classification research, achieving state-of-the-art performance on several benchmark datasets^25–30^. For instance, Najia and Faouzi^27^ proposed an enhanced CNN-BiLSTM architecture with attention mechanisms, achieving strong classification performance; however, the method employs SMOTE-based synthetic oversampling for class imbalance mitigation, which introduces artificially generated samples that may not reflect realistic ECG morphological variations, and the model is evaluated exclusively on MIT-BIH without independent validation on a second dataset. Similarly, the CBGM model^23^, a hybrid architecture combining CNN, BiGRU, and multi-head attention designed for 12-lead ECG classification, reported strong performance on MIT-BIH but demonstrated measurable performance degradation on PTBDB, consistent with the known tendency of models optimized for a single benchmark to overfit to its specific characteristics. However, many existing hybrid CNN-RNN frameworks rely on increasingly complex architectures and are often evaluated primarily on benchmark datasets, limiting understanding of their robustness and generalization across heterogeneous ECG recordings.

In addition to temporal modeling, architectural enhancements such as residual learning and attention mechanisms have been introduced to further improve classification accuracy^31,32^. Residual connections enable the training of deeper networks by facilitating gradient flow and mitigating the vanishing gradient problem, thereby improving feature reuse and model stability^19,33^. Attention mechanisms, on the other hand, allow neural networks to selectively focus on diagnostically relevant portions of the signal, enhancing feature discrimination^34,35^. Recent studies have employed sophisticated attention modules, including multi-head self-attention, to capture global dependencies in ECG signals and achieve impressive performance on widely used datasets^36–39^. However, increasingly sophisticated attention-based architectures often introduce substantial computational complexity and higher memory requirements, which may limit their suitability for lightweight or real-time clinical deployment.

Despite these advances, an important challenge that remains insufficiently addressed is the generalization capability of deep learning models across different ECG datasets. Many existing approaches report exceptionally high accuracy on the MIT-BIH Arrhythmia Database, which has become the de facto benchmark for ECG classification^23,39,40^. While MIT-BIH is invaluable for method development and comparison, it consists of recordings acquired under relatively controlled conditions with limited lead configurations and well-defined beat annotations. As a result, models trained and evaluated exclusively on this dataset may inadvertently overfit to its specific characteristics, leading to overly optimistic performance estimates^41,42^. With the rapid adoption of wearable and ambulatory ECG monitoring devices, vast volumes of long-term ECG recordings are now routinely collected outside clinical environments. These signals are often affected by motion artifacts, noise, and device-specific variations, further increasing data heterogeneity. Automated ECG classification systems must therefore be robust to such variations and capable of generalizing beyond controlled clinical datasets to ensure reliable performance in real-world deployments. This highlights the limitation of relying solely on single-benchmark evaluation, as strong performance on one dataset does not necessarily guarantee robust generalization across heterogeneous real-world ECG distributions.

In contrast, the PTB Diagnostic ECG Database^3^ (PTBDB) presents a more challenging and realistic evaluation scenario. PTBDB contains ECG recordings collected from a larger and more heterogeneous patient population, with variations in acquisition protocols, signal morphology, and pathological conditions^43^. The dataset exhibits higher inter-subject variability, differences in sampling frequency, and diverse ECG patterns across leads, making it substantially different from MIT-BIH in its acquisition and signal characteristics^23^. Consequently, achieving strong performance on PTBDB is widely regarded as a more stringent test of model robustness and generalization capability. Several recent studies have reported lower classification performance on PTBDB compared to MIT-BIH when the same architecture is independently evaluated on each dataset, underscoring the inherently greater difficulty of PTBDB as an evaluation benchmark^23,44,45^. These findings suggest that many existing models remain sensitive to dataset-specific characteristics and may struggle to maintain consistent performance across heterogeneous ECG distributions.

From a clinical perspective, robust generalization is of paramount importance. In real-world healthcare settings, ECG data are acquired using different devices, electrode placements, and recording conditions, introducing substantial heterogeneity across patient populations and clinical environments. A clinically viable automated ECG classification system must therefore demonstrate reliable performance across diverse data distributions rather than excelling on a single curated dataset. This consideration motivates the need for model architectures that balance representational power with regularization and robustness, avoiding unnecessary complexity that may hinder consistent performance across heterogeneous datasets. This study does not aim to introduce architectural complexity for its own sake, nor to replace existing transformer-based or multi-lead fusion approaches. Instead, the focus is on designing a balanced and efficient architecture whose generalizability is demonstrated through independent evaluation on two datasets with fundamentally different classification tasks, class structures, and acquisition characteristics, thereby addressing the key limitation of single-benchmark evaluation that pervades many existing ECG classification models. The generalization capability of the proposed architecture is therefore assessed through independent training and evaluation on each dataset separately, reflecting a rigorous and realistic measure of architectural robustness across heterogeneous ECG data.

Motivated by these challenges, this paper proposes CaReS-BiNet (Convolutional and Residual Squeeze-and-Excitation BiLSTM-based Network), an end-to-end deep learning framework for ECG arrhythmia classification designed to achieve consistent performance across datasets with substantially different acquisition characteristics and classification tasks. The proposed architecture combines the strengths of CNN-based spatial feature extraction and Bidirectional Long Short-Term Memory (BiLSTM)-based temporal modeling while incorporating residual connections to stabilize deep feature learning. Instead of relying on computationally expensive multi-head attention mechanisms, a lightweight Squeeze-and-Excitation (SE) module is employed to adaptively recalibrate channel-wise feature responses. This design choice aims to enhance discriminative feature learning while preserving model simplicity and robustness across heterogeneous ECG datasets. By explicitly integrating BiLSTM-based temporal modeling with convolutional feature extraction, CaReS-BiNet directly addresses the limitation of insufficient long-range temporal dependency modeling inherent in conventional CNN-based ECG classifiers.

The proposed method is systematically evaluated on both the MIT-BIH Arrhythmia Database and the PTBDB through independent training and evaluation on each dataset using their respective data splits. Experimental results demonstrate that CaReS-BiNet achieves competitive performance on MIT-BIH and attains superior accuracy and F1-score on PTBDB compared to recent CNN-RNN-attention-based models, indicating strong and consistent architectural robustness across datasets with substantially different acquisition characteristics and signal distributions. These findings highlight the effectiveness of the proposed architecture in learning robust and dataset-invariant representations, underscoring its potential applicability in real-world clinical environments where ECG data heterogeneity and acquisition variability are inherent.

Our paper takes a novel perspective that sets it apart from existing literature. The main contributions of this study are as follows:

1. Rather than relying on handcrafted features or dataset-specific preprocessing, our work proposes CaReS-BiNet, an end-to-end deep learning architecture that jointly learns local morphological and long-range temporal representations directly from ECG heartbeat segments through the principled integration of multi-scale convolution, residual learning, squeeze-and-excitation attention, and BiLSTM modeling. Each architectural design decision is validated through a systematic ablation study, quantifying the individual contribution of each component across both datasets.
2. Recognizing that most existing models are optimized for a single benchmark, our work explicitly emphasizes generalization by evaluating the proposed architecture independently on three datasets with substantially different acquisition characteristics, classification tasks, and class structures, providing a more rigorous and clinically realistic assessment of architectural robustness.
3. A lightweight Gaussian noise augmentation strategy is used to improve intra-class diversity and reduce overfitting, particularly for underrepresented arrhythmia classes.
4. Binary ventricular ectopic beat (VEB) and supraventricular ectopic beat (SVEB) detection is evaluated following the AAMI binary evaluation protocol, providing a standardised clinical benchmark that complements the five-class evaluation and confirms the clinical reliability of the proposed framework for detecting both life-threatening ventricular arrhythmias and morphologically subtle supraventricular ectopic beats.

Together, these contributions advance the state of automated ECG arrhythmia classification by addressing both the architectural and evaluation-level limitations of existing approaches, with a focus on building a framework that is not only accurate on controlled benchmarks but also consistent across the heterogeneous signal conditions encountered in real-world cardiac monitoring.

## Methods

This section describes the datasets, preprocessing strategy, CaReS-BiNet architecture, training protocol, evaluation metrics, and computational complexity analysis. This section first describes the datasets used for training and evaluation, followed by a detailed presentation of the proposed CaReS-BiNet framework organized into three sequential stages:

1. Signal preprocessing and normalization,
2. Hierarchical feature extraction and temporal modeling, and
3. Classification and performance evaluation.

**Fig. 1:**
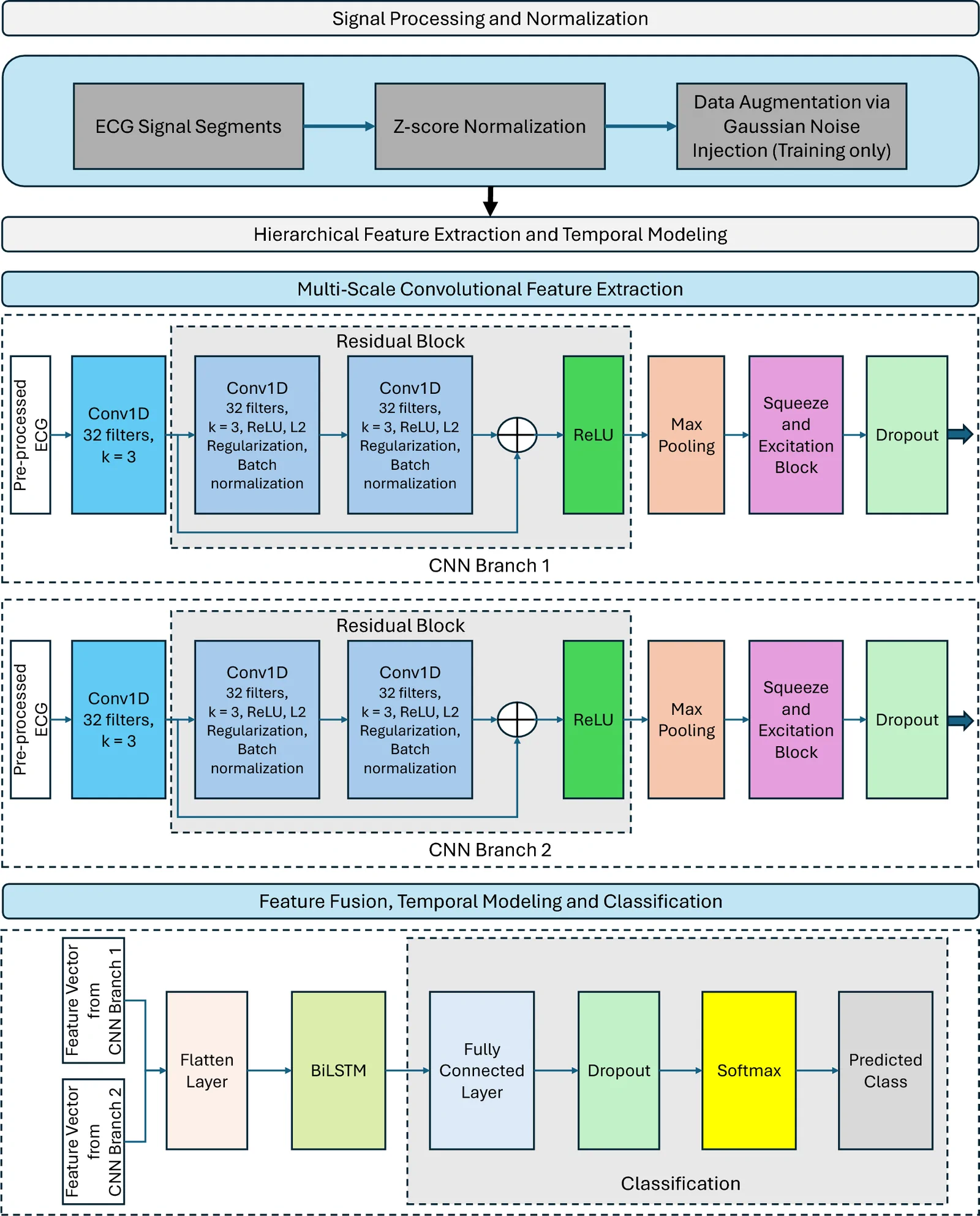
Block diagram of the proposed arrhythmia classification model.

Each stage is intentionally chosen to mitigate specific limitations of existing approaches rather than merely improving classification accuracy on a single dataset. The complete framework is illustrated in Fig. 1.

### Datasets and generalization-oriented evaluation strategy

#### MIT-BIH arrhythmia database

The MIT-BIH Arrhythmia Database is employed as the primary training and evaluation dataset due to its extensive use in prior studies, availability of expert annotations, and standardized evaluation protocols. It consists of 48 half-hour ECG recordings sampled at 360 Hz, with heartbeat segments categorized into five clinically relevant classes under the AAMI EC57 standard: normal beats (N), supraventricular ectopic beats (S), ventricular ectopic beats (V), fusion beats (F), and unclassified beats (Q). The dataset follows a predefined patient-oriented split into two subsets, a training set (DS1) and an independent test set (DS2), ensuring that recordings from the same patient do not appear in both subsets, thereby providing a realistic assessment of inter-patient generalization. While MIT-BIH serves as a reliable benchmark for method development, its relatively controlled acquisition conditions and limited lead configurations make it insufficient for assessing real-world robustness in isolation.

#### PTB diagnostic ECG database (PTBDB)

To explicitly evaluate the architectural robustness of CaReS-BiNet, the PTB Diagnostic ECG Database (PTBDB) is used as an independent evaluation dataset. PTBDB contains ECG recordings from a larger and more heterogeneous patient population, collected under diverse acquisition protocols with varying signal morphologies and pathological conditions. The dataset exhibits significantly higher inter-subject variability compared to MIT-BIH, making it a more stringent and clinically realistic evaluation benchmark. The dataset includes 12 standard leads and 3 Frank leads, sampled at 1000 Hz. It is important to note that CaReS-BiNet is independently trained and evaluated on PTBDB using its own data split, with the output layer adapted to the binary classification task of this dataset.

#### Hybrid single-lead ECG dataset

An additional experiment was conducted using the Hybrid Single-Lead ECG dataset^46^. This dataset is a multi-source ECG dataset derived from the MIT-BIH Arrhythmia, European ST-T, and BIDMC Congestive Heart Failure databases, containing 115,693 single-lead ECG segments with each segment represented by 160 samples and assigned to one of four classes. The labels were integer encoded as Class 0, Class 1, Class 2, and Class 3. To ensure an unbiased evaluation, the dataset was divided using a stratified 80:10:10 split into training, validation, and independent test subsets. CaReS-BiNet was independently trained on this dataset without any dataset-specific architectural modifications, except that the final softmax layer was adjusted to four output classes to match the classification task of this dataset.

### Signal preprocessing and normalization

#### Avoidance of handcrafted segmentation and dataset-agnostic preprocessing

In contrast to many existing approaches^23^ that rely on handcrafted denoising, R-peak detection, or beat segmentation, the proposed framework operates directly on fixed-length ECG heartbeat samples by adopting a minimal preprocessing strategy consisting solely of amplitude normalization using Z-score standardization. This design choice is motivated by two key considerations:

1. Error propagation: R-peak detection errors can propagate downstream and significantly affect classification performance, particularly in noisy or pathological ECG recordings.
2. Generalization: Handcrafted segmentation rules are often tuned to specific datasets and may not perform consistently across databases with different sampling rates, morphologies, or noise characteristics.

By allowing the network to learn discriminative patterns directly from normalized ECG heartbeat samples, the proposed framework avoids reliance on dataset-specific preprocessing heuristics, which is particularly important for maintaining consistent performance when independently evaluating the proposed architecture on PTBDB, where signal characteristics differ significantly from MIT-BIH.

#### Normalization for dataset-invariant learning

To mitigate inter-subject and inter-dataset amplitude variations, ECG signals are normalized using z-score normalization. This step maps signals acquired from different datasets, acquisition systems, and patient populations to a common statistical scale, preventing the learning process from being dominated by amplitude-related biases. Such normalization is particularly important when evaluating the proposed architecture on PTBDB, which differs substantially from the MIT-BIH Arrhythmia Database in sampling rate, lead configuration, and signal dynamic range. By enforcing a consistent input distribution across datasets with differing acquisition characteristics, z-score normalization encourages dataset-invariant feature learning and stabilizes model optimization, thereby directly supporting robust and consistent classification performance across heterogeneous datasets. Unlike batch normalization, which operates on intermediate feature maps during network training, z-score normalization is applied directly to the raw heartbeat samples. Each heartbeat sample is standardized to have zero mean and unit variance, reducing amplitude variability across recordings and facilitating stable and efficient convergence during training.

#### Data augmentation via noise injection

Arrhythmia datasets are inherently imbalanced, with normal sinus beats occurring far more frequently than clinically significant abnormal rhythms. This skewed class distribution biases deep learning models toward majority classes, often resulting in poor sensitivity for minority arrhythmias despite high overall accuracy.

To address these challenges, a Gaussian noise-based data augmentation strategy is employed during training. A lightweight noise injection approach is adopted to simulate realistic perturbations arising from sensor noise, motion artifacts, and acquisition variability, while preserving the underlying physiological characteristics of ECG signals. Empirical studies on time-series classification have shown that simple noise-based augmentation strategies, such as Gaussian jittering, consistently improve model robustness and generalization across diverse signal domains^47^. This strategy increases intra-class variability without altering the morphological integrity of ECG waveforms. Formally, each ECG heartbeat segment *X* is augmented as:

1 $$\begin{aligned} \tilde{X} = X + \mathcal {N}(0, \sigma ^2) \end{aligned}$$

where $\mathcal {N}(0,\sigma ^2)$ denotes Gaussian noise with zero mean and variance $\sigma ^2$. In this study, a small standard deviation of *σ* = 0.01 is used to ensure that the injected noise remains within physiologically plausible limits. The augmented segments are concatenated with the original training samples, effectively increasing the diversity of the training set without altering class labels or morphological structure.

This augmentation strategy serves multiple purposes. First, it mitigates class imbalance by increasing the effective sample diversity of minority arrhythmia classes, thereby reducing bias toward dominant normal beats. Second, it discourages memorization of dataset-specific morphological templates by exposing the network to slight variations of the same underlying beat morphology. Third, it promotes the learning of robust, class-discriminative structural patterns rather than noise-sensitive amplitude details. By improving robustness to signal variability and reducing overfitting, noise-based augmentation directly supports the model’s ability to maintain reliable classification performance across datasets with differing acquisition characteristics. This is particularly important when evaluating the proposed architecture on PTBDB, where signal noise, recording protocols, and patient heterogeneity differ substantially from MIT-BIH. Consequently, the adopted augmentation strategy aligns with the broader objective of developing an architecturally robust and clinically reliable ECG arrhythmia classification framework that maintains consistent performance across datasets with heterogeneous acquisition characteristics.

### Hierarchical feature extraction and temporal modeling

The first stage of the framework focuses on learning discriminative morphological features directly from ECG segments using one-dimensional convolutional layers. Accurate ECG-based arrhythmia classification requires the joint modeling of local morphological characteristics (e.g., QRS complexes, P and T waves) and long-range temporal dependencies that span multiple cardiac cycles. While CNNs are highly effective at extracting local, shift-invariant features, they are inherently limited by their finite receptive fields and thus struggle to capture rhythm-level temporal dependencies. This limitation becomes particularly pronounced for arrhythmias whose diagnostic signatures depend on rhythm irregularities and temporal context rather than isolated waveform shapes. Conversely, recurrent architectures are well suited for sequential modeling but depend on informative feature representations at their input. These observations directly motivate the hierarchical feature extraction and temporal modeling strategy adopted in this work.

#### Multi-scale convolutional feature extraction

The first stage of the framework focuses on learning discriminative morphological features directly from ECG segments using one-dimensional convolutional layers. Each heartbeat sample in the dataset is represented as a fixed-length sequence of amplitude values capturing the morphological profile of a single cardiac cycle. These amplitude sequences exhibit diagnostically relevant patterns at multiple scales: sharp amplitude transitions corresponding to QRS complexes are localised over a small number of consecutive values, whereas broader morphological characteristics such as P-wave and T-wave regions span larger portions of the sequence. Relying on a single convolutional kernel size is therefore insufficient to capture the full spectrum of morphological characteristics encoded in the heartbeat representation. To address this, the proposed architecture employs parallel convolutional branches, each operating on the same normalized heartbeat sample but using different kernel sizes. Each branch applies one-dimensional convolutions with distinct kernel sizes:

- Branch 1 uses a kernel size of 3 to emphasize fine-grained local features, particularly sharp waveform transitions such as QRS complexes.
- Branch 2 uses a kernel size of 5 to capture broader temporal patterns, including waveform morphology and contextual variations spanning larger portions of the heartbeat sequence.

By learning features at multiple temporal scales in parallel, the model avoids reliance on a single fixed receptive field, which is a known limitation of conventional CNN-based ECG classifiers.

Each convolutional branch consists of stacked one-dimensional convolutional layers followed by batch normalization and nonlinear activation functions, enabling stable and efficient feature learning. Batch normalization is employed to reduce internal covariate shift and improve training stability across batches drawn from heterogeneous ECG datasets. Max-pooling layers are applied to progressively reduce temporal dimensionality while introducing translational invariance, thereby reducing sensitivity to minor positional misalignment within heartbeat samples. From a methodological standpoint, this design encourages the network to learn morphology-driven representations that are invariant to small positional shifts and amplitude variations, rather than memorizing dataset-specific amplitude patterns. By extracting local features directly from normalized heartbeat samples and enforcing invariance through pooling, the convolutional component promotes dataset-agnostic feature learning.

#### Residual learning for stable deep feature extraction

Each convolutional branch incorporates residual blocks, in which shortcut connections are introduced between the input and output of stacked convolutional layers. Residual learning facilitates stable gradient propagation and enables effective training of deeper convolutional representations without performance degradation. More importantly, residual connections encourage feature reuse by preserving low-level morphological features and allowing them to be incrementally refined across layers rather than overwritten.

From a methodological perspective, this design improves convergence stability while reducing the tendency of the network to memorize dataset-specific amplitude patterns. By maintaining a consistent flow of physiologically meaningful features through the network, residual learning supports robust feature extraction across datasets with substantially different acquisition characteristics, thereby contributing to consistent architectural performance beyond single controlled benchmarks such as MIT-BIH.

#### Channel-wise feature recalibration via squeeze-and-excitation

Heartbeat samples contain a wide range of morphological characteristics, not all of which are equally informative for arrhythmia diagnosis. Some amplitude patterns carry strong diagnostic relevance, while others may reflect noise, patient-specific variations, or acquisition artifacts. Treating all extracted features as equally important can therefore reduce classification reliability, particularly when heartbeat samples originate from datasets with differing acquisition characteristics. To address this, each convolutional branch integrates a SE block following max-pooling to adaptively emphasize informative features and suppress less relevant ones. Rather than introducing additional handcrafted rules, this mechanism allows the model itself to learn which extracted features are most discriminative for arrhythmia classification.

The SE block first applies global average pooling along the temporal dimension of each feature map. This operation compresses each channel into a single representative value, summarizing its overall contribution across the entire ECG segment. These channel-wise summaries are then passed through a small gating network composed of two fully connected (dense) layers. The first dense layer reduces the channel dimensionality using a ReLU activation, enabling the model to learn compact inter-channel relationships. The second dense layer restores the original number of channels and applies a sigmoid activation function, producing a set of normalized values between 0 and 1. Based on this summary, the network learns a set of channel-wise weights, referred to as learned gates, that quantify the relative importance of each feature channel. Values close to 1 indicate highly informative channels, whereas values close to 0 suppress channels that contribute little to the classification task. These learned gate values are reshaped and multiplied with the original feature maps, resulting in adaptive rescaling of each channel. As a result, channels carrying clinically meaningful ECG patterns are amplified, while less relevant features are attenuated.

By allowing the network to focus selectively on informative ECG features, the SE mechanism improves robustness to noise, inter-subject variability, and dataset-specific characteristics.

#### Regularization and overfitting control

To mitigate the risk of overfitting the model with data set-specific features, the proposed framework incorporates multiple architectural and optimization-level regularization strategies that constrain model complexity while preserving discriminative power.

Dropout is applied within the convolutional branches, recurrent layers, and fully connected layers to prevent excessive co-adaptation of neurons. By randomly disabling a subset of activations during training, dropout forces the network to rely on distributed feature representations rather than a small set of highly specialized neurons. This improves robustness to noise and variability, reduces sensitivity to dominant normal rhythm patterns, and enhances minority-class generalization. In addition, L2 weight regularization is applied to convolutional and dense layers to penalize overly complex parameter configurations, promoting smoother and more generalizable feature representations. Batch normalization further stabilizes intermediate feature distributions and improves convergence, collectively reducing overfitting and supporting reliable performance on different datasets.

#### Feature fusion across scales

The outputs of the two convolutional branches are flattened and concatenated, forming a unified multi-scale feature representation. This fusion step allows the model to integrate both fine-resolution and coarse-resolution features before temporal modeling. The merged feature vector is then reshaped to serve as input to the temporal modeling.

#### Temporal modeling with BiLSTM

After multi-scale feature fusion, the combined feature representation is passed to a BiLSTM network for sequential modeling of within-segment dependencies. While the convolutional branches effectively extract local morphological patterns from individual heartbeat samples, the feature maps produced by convolutional layers retain a sequential structure corresponding to different positional regions of the heartbeat amplitude sequence. Modeling the dependencies across these positional feature representations is therefore essential for capturing higher-level morphological relationships that local convolution alone cannot fully characterize.

The BiLSTM network processes the fused feature sequence in both forward and backward directions, enabling the model to consider contextual information from earlier and later portions of the heartbeat amplitude sequence simultaneously. This bidirectional processing enhances the network’s ability to identify subtle morphological patterns whose diagnostic significance depends on their relationship to surrounding amplitude regions, for example, the shape of the ST segment relative to the QRS complex, or the relationship between P-wave and T-wave regions within the same heartbeat. By integrating multi-scale convolutional feature extraction with bidirectional sequential modeling, the proposed framework captures both fine-grained local amplitude patterns and broader intra-segment morphological relationships, leading to a more comprehensive and discriminative heartbeat classification strategy.

### Classification and performance evaluation

The temporally enriched feature representation produced by the BiLSTM is passed to fully connected layers for final arrhythmia classification. While the BiLSTM captures sequential dependencies and rhythm-level information, the dense layers transform these learned representations into a compact and discriminative feature space suitable for class prediction. A fully connected layer with nonlinear activation is first applied to further refine the high-level temporal features. This layer enables the model to combine morphological and rhythm-based information into a unified decision representation. Dropout was applied before the final classifier to reduce overfitting. The final output layer uses a softmax activation function to generate probability scores corresponding to each arrhythmia class. The class with the highest predicted probability is selected as the final diagnosis. This probabilistic formulation allows the model to provide confidence estimates alongside predictions, which is particularly valuable in clinical decision-support settings.

The model is trained using mini-batch gradient-based optimization with the Adam optimizer to ensure stable and efficient convergence. Sparse categorical cross-entropy is used as the loss function, which is appropriate for multi-class classification with integer-encoded labels. A validation subset is used to monitor performance during training, and early stopping is applied to prevent overfitting by restoring the best-performing model based on validation loss. A learning rate reduction strategy is also implemented to adjust the optimization process when validation performance plateaus, thereby improving convergence stability.

To assess performance, the trained model is evaluated on unseen test data without further fine-tuning. Standard classification metrics including accuracy, precision, recall, and F1-score are reported to provide a balanced evaluation across majority and minority arrhythmia classes. These metrics ensure that performance assessment reflects not only overall correctness but also the model’s ability to detect clinically important abnormal rhythms. This structured training and evaluation protocol supports robust performance estimation and consistent architectural generalizability across heterogeneous datasets with different acquisition characteristics and classification tasks.

It is important to note that CaReS-BiNet is independently trained and evaluated on each dataset using their respective data splits, with the output layer adapted to the number of classes in each dataset. The datasets differ not only in the number of output classes but also in the semantic nature of those classes. MIT-BIH requires beat-level morphological discrimination across five AAMI arrhythmia categories, whereas PTBDB involves binary classification of broader cardiac pathological conditions, making direct weight transfer between datasets less meaningful within the scope of this study. The generalization capability assessed in this work therefore refers to the ability of the proposed architecture to maintain applicability across multiple heterogeneous ECG datasets with fundamentally different classification tasks, rather than direct weight transfer between datasets. In addition to MIT-BIH and PTBDB, the proposed framework is further evaluated on an additional heterogeneous ECG dataset to provide a broader assessment of architectural robustness across diverse ECG classification scenarios.

### Computational complexity analysis

To assess the computational efficiency of the proposed CaReS-BiNet model, model complexity was quantified in terms of total parameters, trainable parameters, non-trainable parameters, floating-point operations (FLOPs), and inference latency. FLOPs were estimated analytically on a layer-wise basis for a single ECG beat segment using the convention that one multiply and one addition correspond to two FLOPs. Convolutional, dense, and BiLSTM layers were included in the FLOP estimation, whereas dropout, Gaussian noise, flattening, reshaping, and concatenation layers were excluded because they do not contribute meaningful arithmetic operations during inference. Inference latency was measured under inference mode after warm-up runs using both single-beat and batch-wise prediction settings. Batch-wise inference was evaluated with a batch size of 32 to estimate practical throughput during ECG screening.

## Results and discussion

### Experimental setup

The proposed model was first evaluated using the MIT-BIH Arrhythmia Database to assess its effectiveness in multi-class ECG heartbeat classification. The dataset consists of annotated heartbeat segments representing multiple arrhythmia categories. The MIT-BIH Arrhythmia Database consists of two predefined subsets: a training set and an independent test set. The training set is further partitioned to reserve 10% for validation. The validation subset was used during training to monitor generalization performance and guide early stopping, while the independent test set was kept completely unseen until final evaluation to ensure unbiased performance assessment.

The model was trained for a maximum of 100 epochs with a batch size of 32 using the Adam optimizer with an initial learning rate of 0.0005. A learning rate scheduling strategy was applied to automatically reduce the learning rate when validation loss plateaued, facilitating stable convergence. Early stopping was applied based on validation loss to prevent overfitting, and the best-performing model weights were restored automatically. Final performance metrics were computed exclusively on the independent test set to report classification performance.

### Quantitative performance on the MIT-BIH arrhythmia database

To obtain deeper insight into classification behavior beyond overall accuracy, precision, recall, and F1-score were computed for each arrhythmia class on the independent test set of the MIT-BIH Arrhythmia Database. The proposed model achieved a training accuracy of 98.76% and a validation accuracy of 98.64%, indicating stable convergence during training with minimal performance fluctuation between training and validation sets. On the independent test set, the model attained an overall accuracy of 98.74%, which is slightly higher than the validation performance and consistent with the training results.

**Fig. 2:**
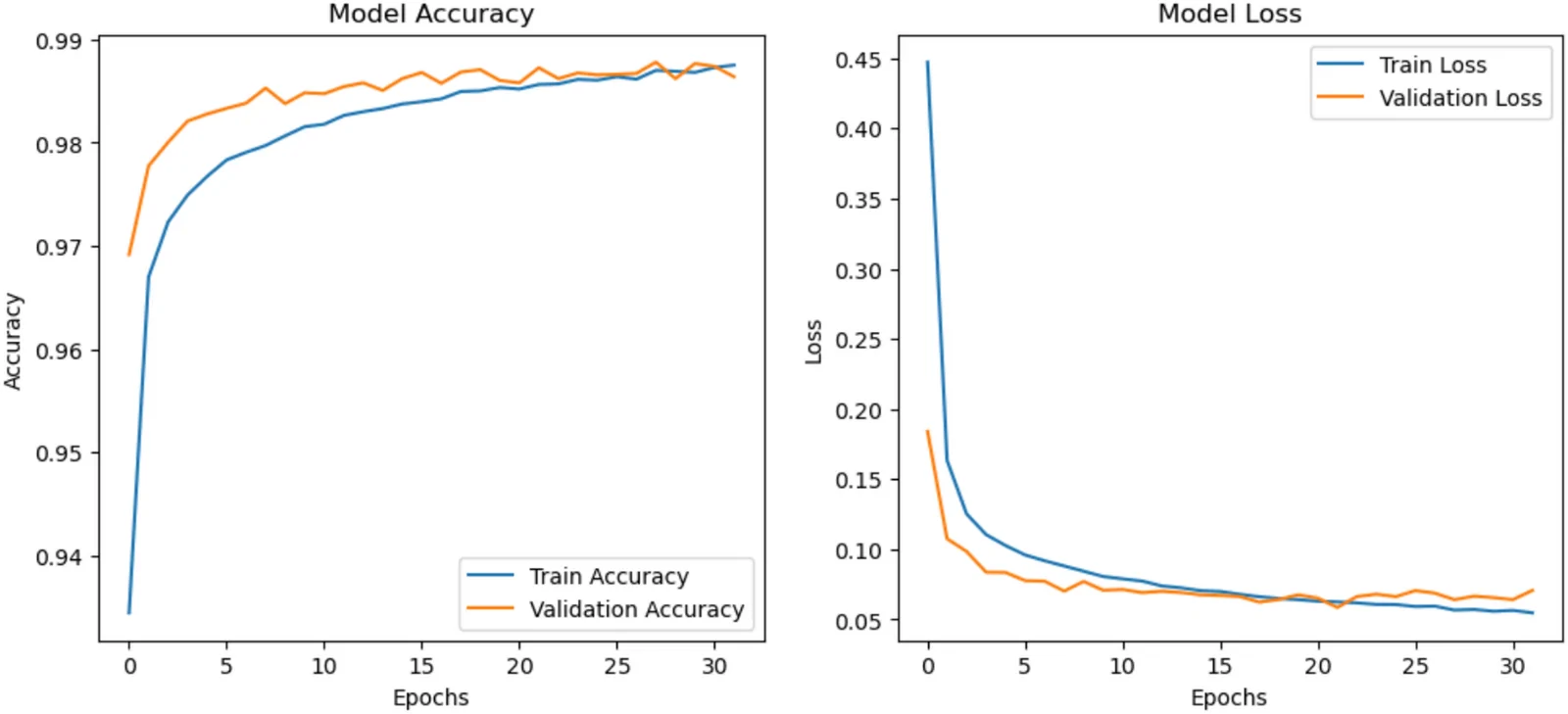
Training and validation accuracy and loss curves of the proposed model on the MIT-BIH database.

A comparison between the training and validation accuracy and loss curves is presented in Fig. 2 to analyze the convergence behavior of the proposed model. The validation accuracy increases progressively alongside the training accuracy throughout the training process, demonstrating stable learning dynamics. The final training and validation accuracies reach 98.76% and 98.64%, respectively, reflecting a very small difference of approximately 0.12%. The close alignment between the two curves, metrics, and the absence of sharp divergence between the loss curves indicate that the model generalizes well to unseen validation data without exhibiting significant overfitting or underfitting. Similarly, the validation loss follows a trend similar to the training loss, decreasing steadily and stabilizing as training progresses. Furthermore, the consistent test accuracy (98.74%) confirms that the model maintains its predictive capability when evaluated on completely unseen data, thereby demonstrating strong generalization performance and validating the effectiveness of the proposed architecture. Overall, the results demonstrate that the proposed architecture achieves both high accuracy and stable generalization, making it suitable for reliable automated arrhythmia classification.

**Fig. 3:**
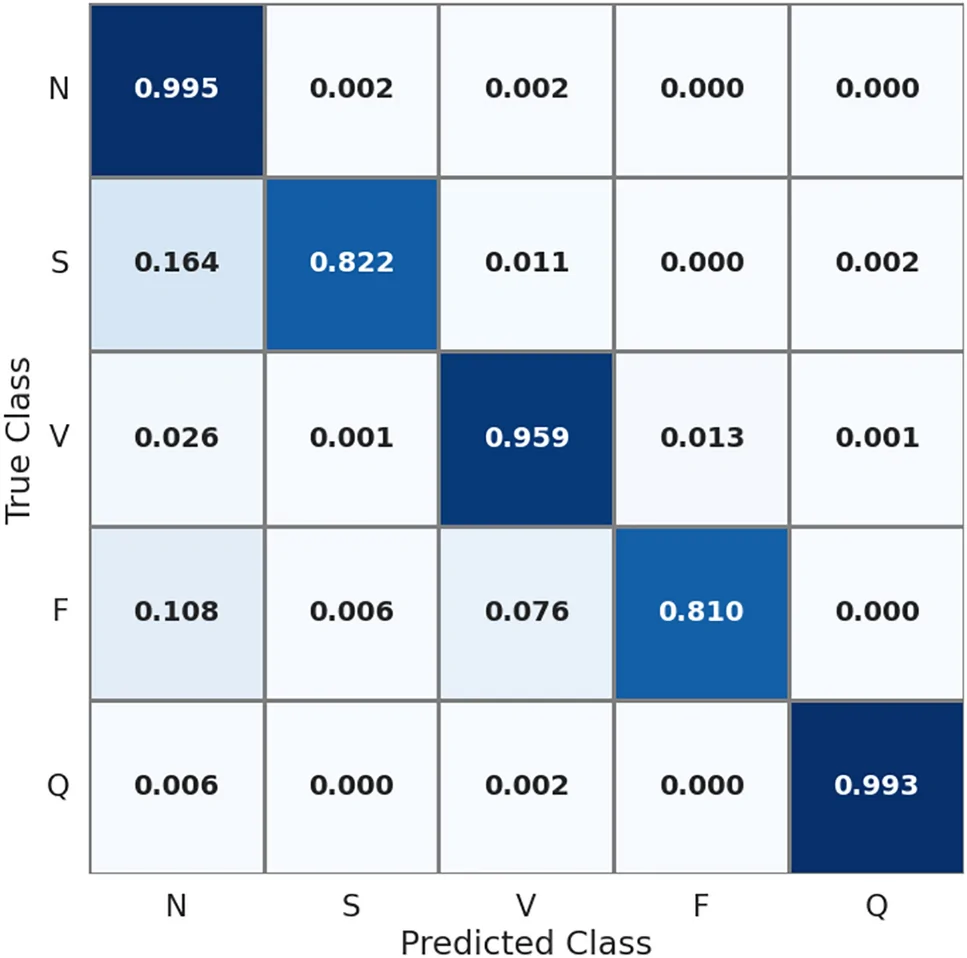
Normalized confusion matrix for the MIT-BIH test set.

The normalized confusion matrix for the MIT-BIH test set is presented in Fig. 3, where diagonal entries represent the correctly classified proportions for each AAMI class. The model demonstrates near-perfect classification for the N class, achieving a recall of 99.5%, which reflects both the abundance of normal beats in the training distribution and the model’s ability to learn highly discriminative morphological features for sinus rhythm. The Q class similarly attains a recall of 99.3%, indicating reliable detection of paced and unknown beats despite their distinct waveform characteristics. The V class (ventricular ectopic beats) achieves a recall of 95.9%, with minor confusion directed toward the F class (1.3%), which is physiologically expected given that fusion beats share partial ventricular morphology. While the proposed model achieves strong overall classification performance of 98.74%, a per-class analysis reveals the persistent challenge of minority class detection under AAMI grouping. The S class (supraventricular ectopic beats) yields a recall of 82.2%, with the dominant source of error being misclassification as N (16.4%). This confusion is clinically interpretable, as supraventricular beats often closely resemble normal sinus morphology in single-lead recordings, differing primarily in subtle P-wave characteristics and timing that are difficult to resolve from short fixed-length segments. The F class (fusion beats) exhibits the lowest recall at 81.0%, with notable confusion toward N (10.8%) and V (7.6%), consistent with the inherent morphological ambiguity of fusion beats, which by definition represent hybrid waveforms arising from simultaneous activation of ventricular tissue by both supraventricular and ectopic impulses. Collectively, these results confirm that classification errors are not random but follow clinically meaningful patterns, with misclassifications occurring predominantly between morphologically similar beat types, a finding well-documented across the ECG classification literature^5,24,48^. The adopted Gaussian noise augmentation strategy, while effective at improving generalization across heterogeneous datasets and stabilizing convergence, as further evidenced in the architectural robustness evaluation presented in the PTBDB results subsection, provides only partial mitigation of extreme class imbalance, as simple noise injection cannot fully compensate for the fundamental scarcity of F and S beats in the training distribution. Future work may explore class-weighted loss functions, focal loss, or oversampling strategies such as SMOTE to further improve minority class sensitivity without sacrificing overall accuracy.

**Fig. 4:**
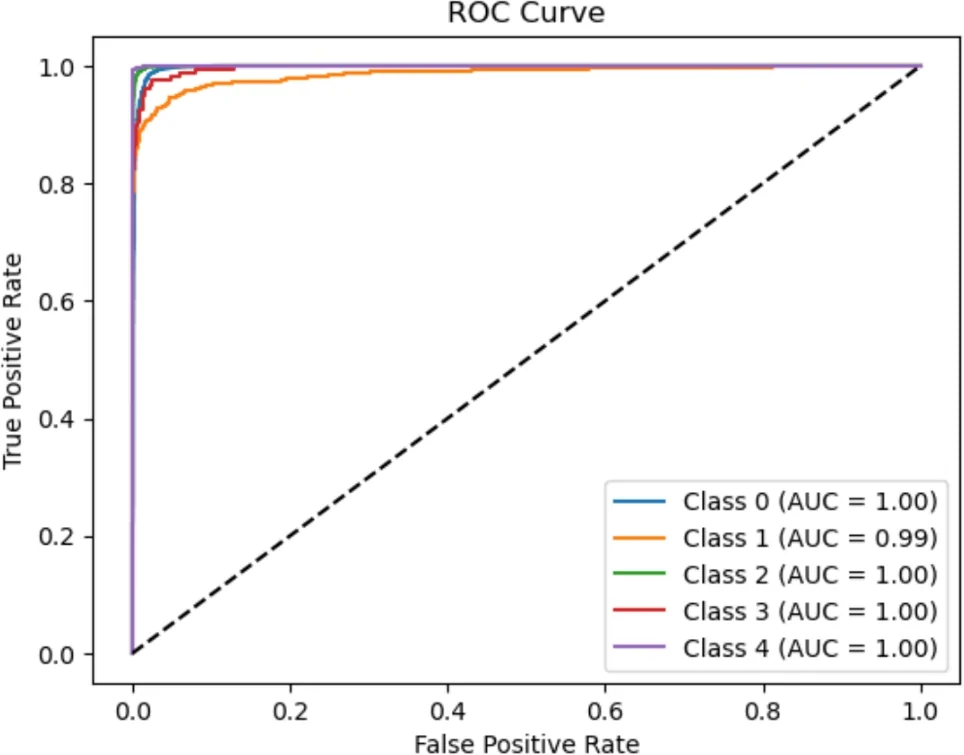
ROC curves for all five AAMI classes on the MIT-BIH test set.

The Receiver Operating Characteristic (ROC) curves for all five AAMI classes are presented in Fig. 4, with the corresponding Area Under the Curve (AUC) values quantifying the model’s discriminative ability at varying classification thresholds. The N (Class 0), V (Class 2), F (Class 3), and Q (Class 4) classes each attain a perfect AUC of 1.00, indicating that the model can achieve flawless separation between these classes and the rest at some operating threshold, regardless of the trade-off between sensitivity and specificity. The S class (Class 1) achieves an AUC of 0.99, which, while marginally lower, still reflects excellent discriminative performance. The slight deviation from unity for the S class is consistent with the confusion matrix findings, where 16.4% of supraventricular beats were misclassified as N, reflecting the morphological proximity of these two classes in the learned feature space. Notably, the near-perfect AUC values across all classes demonstrate that the model’s probabilistic outputs are well-calibrated and highly separable at the score level, even for minority classes whose recall values are comparatively lower. This distinction between AUC and recall is important: high AUC confirms that the model assigns higher confidence scores to correct predictions, while lower recall for S and F classes reflects the challenge of selecting an optimal decision boundary under severe class imbalance.

**Fig. 5:**
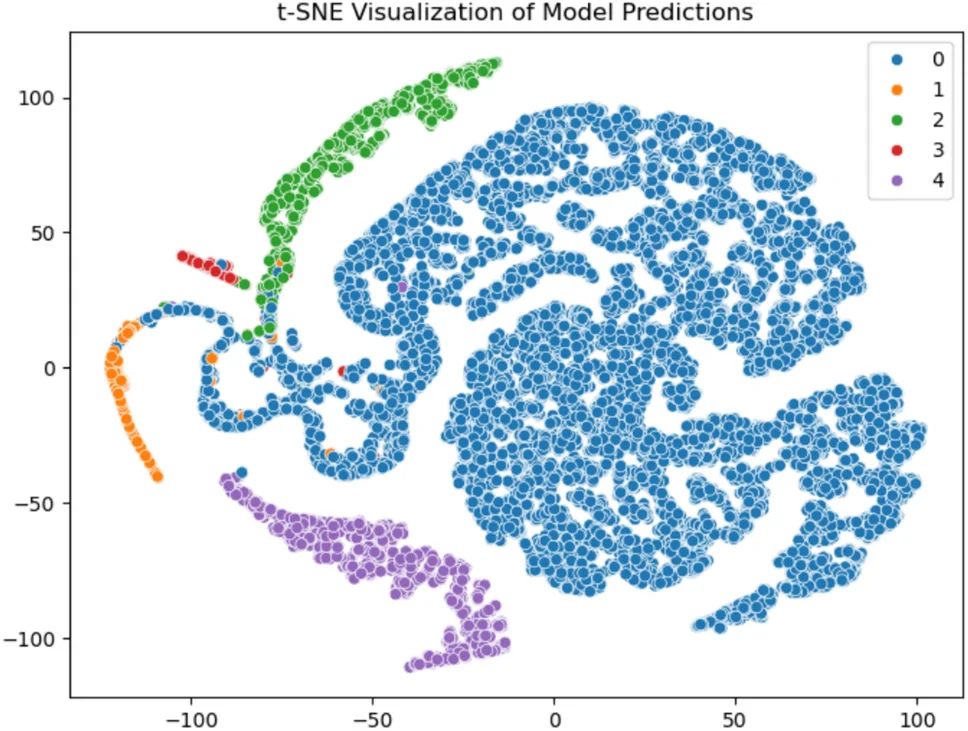
t-SNE visualization of learned feature representations on the MIT-BIH test set.

The t-SNE visualization of the model’s learned feature representations on the MIT-BIH test set is presented in Fig. 5, providing qualitative insight into the separability of the extracted feature embeddings across AAMI classes. The N class (blue) occupies a large, densely clustered region in the center-right of the embedding space, consistent with its dominance in the dataset and the high intra-class diversity of normal sinus beats. The V class (green) and Q class (purple) form compact, well-separated clusters in distinct regions of the embedding space, confirming that the model has learned highly discriminative representations for these classes, consistent with their strong recall values of 95.9% and 99.3%, respectively. The S class (orange) forms an elongated, relatively coherent cluster but exhibits partial overlap with the boundary of the N class region, which directly explains the 16.4% misclassification rate of S beats as N observed in the confusion matrix. The F class (red) appears as a small, loosely distributed cluster in close proximity to both the N and V regions, reflecting the morphological ambiguity of fusion beats and their tendency to be confused with both classes. Overall, the t-SNE visualization corroborates the quantitative results, demonstrating that the proposed model learns geometrically meaningful and largely well-separated feature representations, with residual overlap confined to the clinically expected boundary regions between morphologically similar beat types.

### Performance comparison with state-of-the-art methods

**Table 1:** Performance comparison with state-of-the-art ECG arrhythmia classification methods on the MIT-BIH arrhythmia database under the AAMI EC57 five-class protocol.

Method	Accuracy (%)	Precision (%)	Recall (%)	F1-Score (%)
Alvarado et al.^5^	93.60	53.50	71.86	57.38
Aphale et al.^16^	92.73	91.00	92.00	91.00
Oliveira et al.^18^	95.30	50.04	53.34	51.37
Kiranyaz et al.^15^	96.60	–	67.37	–
Qurraei et al.^6^	98.60	88.07	80.31	81.78
Irid et al.^24^	98.17	–	94.17	–
Najia and Faouzi^27^	99.20	–	97.50	98.29
Proposed CaReS-BiNet	98.74	98.70	98.73	98.71

– indicates the metric was not reported under the AAMI five-class evaluation protocol

Table 1 compares the proposed CaReS-BiNet model against seven existing methods for ECG arrhythmia classification, all evaluated on the MIT-BIH Arrhythmia Database following the AAMI EC57 standard that defines five heartbeat categories: N, S, V, F and Q. Performance is reported using metrics, which account for class distribution across the dataset and provide a comprehensive measure of classification performance across all five classes. The proposed model achieves an accuracy of 98.74%, precision of 98.70%, recall of 98.73%, and F1-score of 98.71%, outperforming the majority of compared methods across all reported metrics. Methods reporting incomplete metrics are included for completeness, with blank entries indicating figures not explicitly reported by the respective authors.

The performance gap between the proposed model and competing methods becomes particularly evident when examining how each method handles the less frequent but clinically critical arrhythmia classes. Methods such as Oliveira et al.^18^ and Alvarado et al.^5^, despite reporting reasonable overall accuracies of 95.30% and 93.60% respectively, reveal severe weaknesses in minority class detection through their low precision values of 50.04% and 53.50% and recall values of 53.34% and 71.86%, indicating that roughly half of their arrhythmia predictions are incorrect and a large proportion of actual arrhythmia beats go undetected. Qurraei et al.^6^ achieve a comparable accuracy of 98.60% through sophisticated time-frequency signal analysis, yet their precision of 88.07% and recall of 80.31% remain notably below the proposed model, reflecting the limitations of handcrafted feature extraction methods that cannot adaptively learn the subtle morphological distinctions between arrhythmia classes the way deep learning architectures can. Kiranyaz et al.^15^ employ a personalised approach where a separate model is trained for each individual patient, achieving a recall of 67.37%. While this patient-specific strategy can capture individual heartbeat patterns, it requires labelled data and retraining for every new patient, making it impractical for large-scale automated screening, a limitation the proposed CaReS-BiNet model entirely avoids by training once on a representative population and generalising across all patients. Aphale et al.^16^ achieve competitive metrics through aggressive data augmentation and the use of an additional database beyond the standard evaluation protocol, conditions that are not available under the standard comparison framework and therefore represent an advantaged experimental setup relative to the proposed model.

Among the most competitive methods, Irid et al.^24^ report an accuracy of 98.17% and recall of 94.17%, but do not report precision or F1-score, precluding a complete comparison. Najia and Faouzi^27^, the most recent method in this comparison, report the highest accuracy of 99.20% and F1-score of 98.29% through a CNN-CBAM-BiLSTM architecture combined with SMOTE oversampling. While their accuracy marginally exceeds the proposed model by 0.47 percentage points, the proposed model achieves a superior weighted recall of 98.73% compared to their recall of 97.50% and a higher F1-score of 98.71% compared to their 98.29%, demonstrating better overall balance between correctly detecting arrhythmias and avoiding false predictions. Furthermore, Najia and Faouzi rely on SMOTE to synthetically generate minority class samples during training, which artificially augments the training distribution and can produce optimistic performance estimates that may not generalise to real-world clinical data where such synthetic samples are unavailable. The proposed model, by contrast, achieves competitive and in key metrics superior performance without synthetic oversampling, demonstrating a more robust and naturally generalisable classification capability. Collectively, these results confirm that the proposed architecture delivers state-of-the-art performance characterised by the best recall and F1-score among all compared methods, making it well-suited for reliable and balanced arrhythmia detection in clinical practice.

**Table 2:** Performance comparison for VEB and SVEB detection on the MIT-BIH arrhythmia database.

Method	VEB (V vs. Rest)			SVEB (S vs. Rest)		
	Accuracy (%)	Precision (%)	Recall (%)	Accuracy (%)	Precision (%)	Recall (%)
Alvarado et al.^5^	–	94.82	92.43	–	56.68	86.19
Kiranyaz et al.^15^	–	96.20	95.90	–	79.20	68.80
Proposed CaReS-BiNet	99.30	96.99	97.38	94.64	97.99	94.64

– indicates the metric was not reported by the respective authors

While the five-class AAMI EC57 classification provides a comprehensive evaluation of overall arrhythmia detection capability, the AAMI standard also specifically recommends evaluating classifiers on two clinically critical binary detection tasks: Ventricular Ectopic Beat (VEB) detection, which distinguishes V-class beats from all other beats (N, S, F, Q), and Supraventricular Ectopic Beat (SVEB) detection, which distinguishes S-class beats from all remaining classes (N, V, F, Q). This binary evaluation framework is clinically motivated. Ventricular ectopic beats are associated with potentially life-threatening conditions such as ventricular tachycardia and fibrillation, where missed detections can have serious consequences for patient safety, while supraventricular ectopic beats are related to atrial fibrillation and other disorders of the atrial rhythm that require timely clinical intervention. Reporting binary VEB and SVEB metrics also enables direct comparison with a wider body of literature that evaluates classifiers specifically on these two tasks, providing a standardised benchmark that complements the five-class evaluation. Table 2 presents the binary classification performance of the proposed model against Alvarado et al.^5^ and Kiranyaz et al.^15^, the two methods in this comparison that explicitly report VEB and SVEB metrics under the AAMI binary evaluation protocol.

In VEB detection, the proposed model achieves an accuracy of 99.30%, precision of 96.99%, and recall of 97.38%, outperforming Alvarado et al. on both precision (94.82%) and recall (92.43%), and remaining competitive with Kiranyaz et al. who report a precision of 96.20% and recall of 95.90%. The marginal difference between the proposed model and Kiranyaz et al.^15^ on VEB metrics is particularly noteworthy given that Kiranyaz et al.^15^ employ a patient-specific paradigm, training a dedicated model per patient using labelled data from that individual, which provides an inherent advantage in capturing patient-specific ventricular morphology. The proposed model, trained once on the population-level dataset and generalising across all patients, achieves comparable VEB detection performance without any patient-specific retraining, demonstrating the effectiveness of its hierarchical convolutional and temporal architecture in learning generalisable ventricular ectopic beat representations.

The SVEB detection results reveal an even stronger advantage for the proposed model. The proposed model achieves a precision of 97.99% and recall of 94.64%, substantially outperforming both Alvarado et al. (precision 56.68%, recall 86.19%) and Kiranyaz et al.^15^ (precision 79.20%, recall 68.80%). The precision improvement over Alvarado et al.^5^ of 41.31 percentage points is particularly significant, their low SVEB precision of 56.68% indicates that more than four out of every ten predicted S-class beats are false alarms, a clinically unacceptable false positive rate for supraventricular ectopic beat screening. Kiranyaz et al.^15^ achieve a higher precision of 79.20% but their recall of 68.80% reveals that nearly one in three actual SVEB beats goes undetected, limiting clinical utility. The proposed model achieves both high precision and high recall simultaneously for SVEB detection, confirming that the BiLSTM temporal modelling combined with squeeze-and-excitation attention provides the discriminative capacity necessary to reliably distinguish supraventricular ectopic beats from normal beats, a classification boundary that competing methods consistently struggle to learn due to the morphological similarity between S-class and N-class beats. Collectively, the binary classification results reinforce the five-class findings and confirm that the proposed model delivers clinically reliable detection performance for both the most dangerous ventricular arrhythmias and the more morphologically subtle supraventricular arrhythmias.

### Architectural robustness evaluation on the PTB diagnostic ECG database

To further evaluate the architectural robustness of the proposed framework, the model was trained and evaluated independently on the PTBDB following the same preprocessing pipeline and architectural configuration described in the Methods section, without any dataset-specific modifications. The PTBDB consists of two predefined subsets: a training set and an independent test set. The training set is further partitioned to reserve 10% for validation following the same protocol adopted for MIT-BIH, while the test set was kept completely unseen until final evaluation. This experiment serves to validate whether the proposed architecture and preprocessing strategy remain effective when applied to a dataset with substantially different characteristics, including higher sampling rate, greater patient heterogeneity, and a binary classification structure, compared to the five-class AAMI setup of MIT-BIH.

**Fig. 6:**
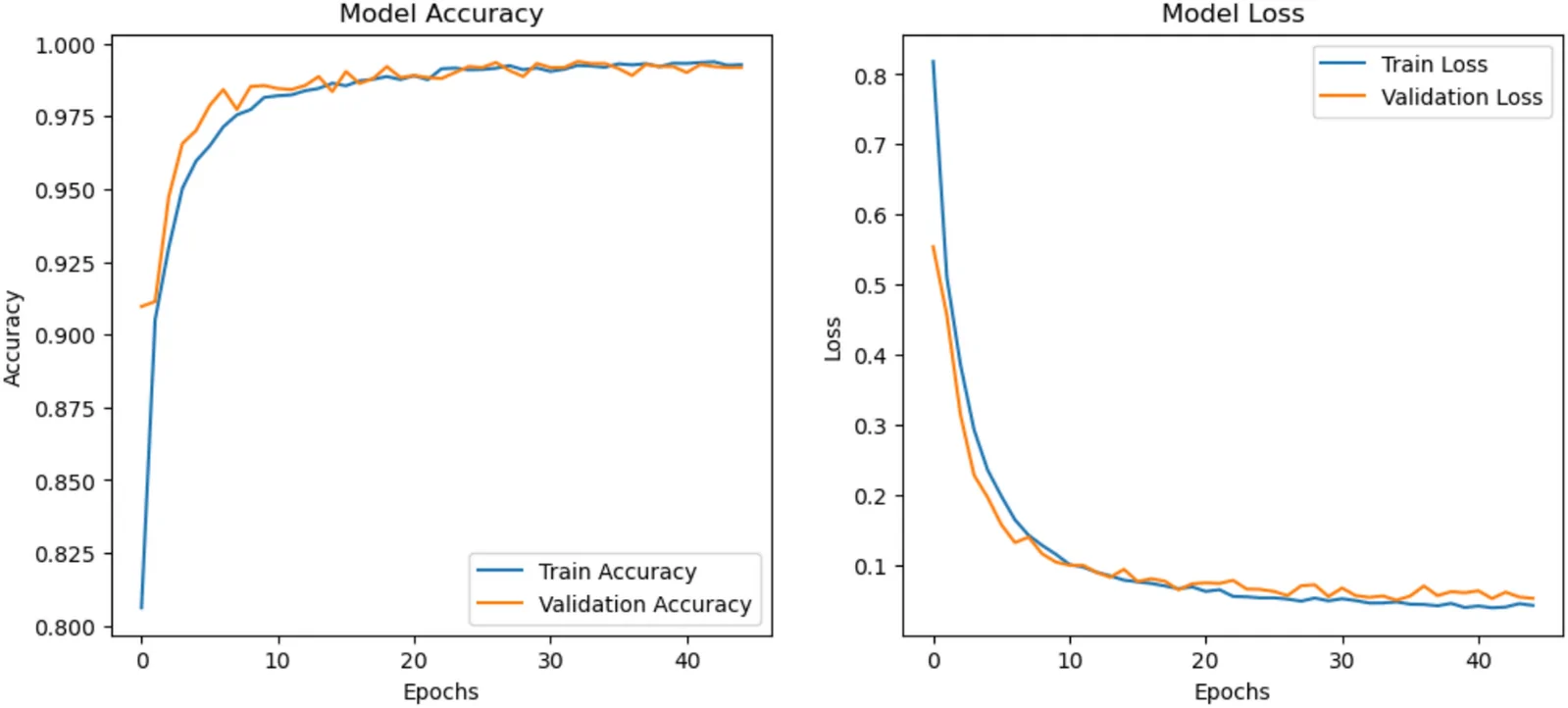
Training and validation accuracy and loss curves of the proposed model on the PTBDB dataset.

The model achieved an overall accuracy of 99.31% on the PTBDB test set, demonstrating that the proposed architecture maintains strong and consistent classification performance on a dataset with substantially different acquisition characteristics, class structure, and classification task without requiring any architectural redesign or dataset-specific tuning. The training and validation accuracy and loss curves presented in Fig. 6 confirm stable convergence, with both curves aligning closely throughout training and stabilizing above 99% by approximately epoch 25. The absence of divergence between training and validation trajectories indicates that the regularization strategies embedded in the architecture, including dropout, L2 weight decay, and batch normalization, remain effective in preventing overfitting on the PTBDB training distribution.

**Fig. 7:**
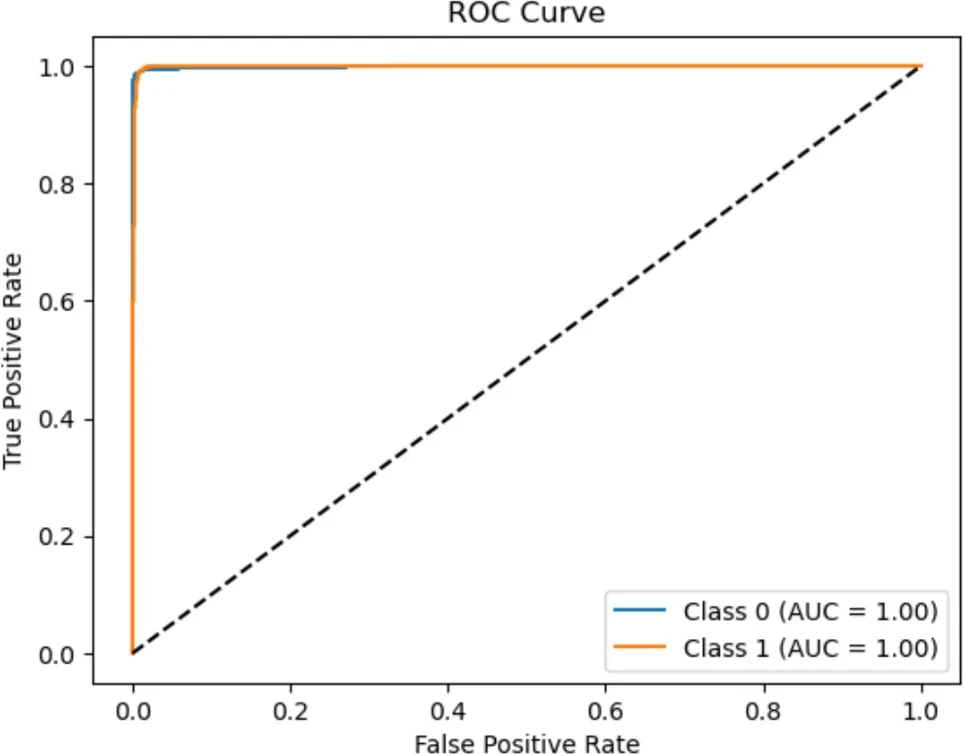
ROC curves for two classes on the PTBDB test set.

**Fig. 8:**
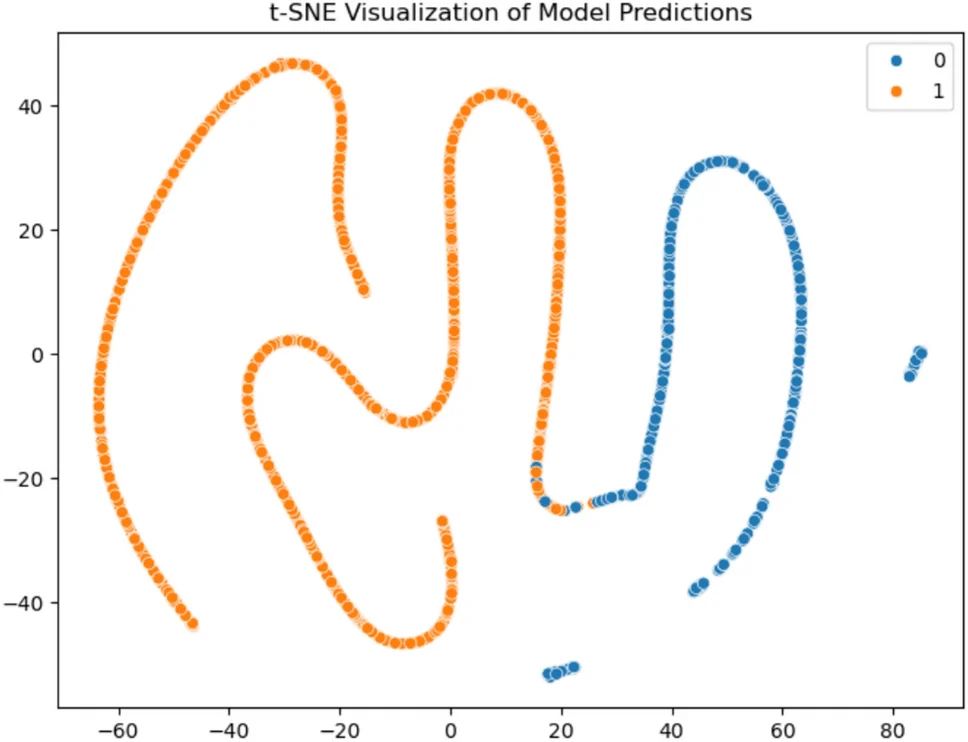
t-SNE visualization of learned feature representations on the PTBDB test set.

The per-class classification performance further supports these findings. The Normal class (Class 0) achieves a precision of 99.15%, recall of 98.44%, and F1-score of 98.80%, while the Abnormal class (Class 1) attains a precision of 99.38%, recall of 99.66%, and F1-score of 99.52%. The marginally lower recall for the Normal class (98.44%) suggests that a small proportion of normal recordings are conservatively classified as abnormal, a clinically preferable error direction that minimizes the risk of missing true pathological cases. The average F1-score of 99.16% confirms balanced and robust performance across both classes, demonstrating that the model does not disproportionately favor the larger Abnormal class despite the inherent class imbalance in the test set (834 normal vs. 2077 abnormal samples).

The ROC curves presented in Fig. 7 demonstrate near-perfect AUC values of 0.999 for both classes, which rounds to 1.00 as displayed in the figure, with curves rising sharply toward the top-left corner at near-zero false positive rates, confirming excellent threshold-independent discriminative performance across both classes. This confirms that the model’s probabilistic outputs are highly separable across all classification thresholds, reflecting excellent discriminative ability for both classes. The t-SNE visualization presented in Fig. 8 further corroborates these results, revealing well-separated and geometrically coherent clusters for the Normal and Abnormal classes in the learned feature embedding space. The Abnormal class forms a continuous curvilinear manifold across the left portion of the embedding space, while the Normal class occupies a compact arc-shaped region on the right, with minimal inter-class boundary overlap. A small number of peripheral points correspond to the marginal misclassifications reflected in the 98% recall of the Normal class. Collectively, these results demonstrate that the proposed framework achieves strong and consistent performance across both MIT-BIH and PTBDB, confirming that the learned representations remain discriminative and well-separated across datasets with substantially different acquisition conditions, class structures, and patient populations.

**Table 3:** Independent evaluation performance comparison on the PTB diagnostic ECG Database.

Method	Accuracy (%)	Precision (%)	F1-Score (%)	AUC
CBGM^23^	98.82	98.33	97.31	0.987
Proposed (CaReS-BiNet)	99.31	99.27	99.16	0.999

To contextualise the independent evaluation performance of CaReS-BiNet on PTBDB, its results are compared against the recently proposed CBGM model^23^, a deep learning architecture combining CNN, BiGRU, and multi-head attention modules. This comparison is particularly relevant for two reasons. First, the CBGM model explicitly evaluates performance on PTBDB, making it one of the few existing methods that reports results on this more challenging dataset, thereby enabling a direct and meaningful comparison. Second, the CBGM model reports strong performance on the MIT-BIH dataset, yet its PTBDB accuracy of 98.82% reflects a measurable performance degradation compared to its MIT-BIH results, a pattern consistent with the known difficulty of maintaining consistent performance across datasets with differing acquisition characteristics, sampling rates, and patient populations.

Table 3 presents the comparative results. CaReS-BiNet achieves an accuracy of 99.31%, precision of 99.27%, F1-score of 99.16%, and an AUC of 0.999 on the PTBDB test set, outperforming the CBGM model across all reported metrics. PTBDB is widely regarded as a more challenging and clinically realistic dataset than MIT-BIH, exhibiting greater inter-subject variability, higher sampling rate, diverse pathological conditions, and substantially different beat morphology, factors that collectively make it a more stringent evaluation benchmark. The fact that CaReS-BiNet not only maintains but improves its performance on PTBDB compared to MIT-BIH, achieving 99.31% accuracy against 98.74% on MIT-BIH, demonstrates that the proposed architecture is capable of learning relevant and discriminative representations from datasets with substantially different acquisition characteristics, class structures, and classification tasks, confirming the architectural flexibility and robustness of the proposed design rather than its dependence on any single dataset’s specific characteristics. Notably, the F1-score improvement of 1.85 percentage points over CBGM’s 97.31% is particularly significant, as F1-score reflects balanced performance between precision and recall and is a more robust indicator of classification quality than accuracy alone under class-imbalanced conditions. The AUC of 0.999 confirms near-perfect threshold-independent discriminative performance, substantially exceeding CBGM’s 0.987 and demonstrating that CaReS-BiNet maintains excellent separation between normal and abnormal classes across all operating thresholds, as further evidenced by the ROC curves presented in Fig. 7. The ability of CaReS-BiNet to outperform a multi-lead architecture on this challenging dataset provides strong evidence that principled architectural design choices - specifically the integration of multi-scale convolutional feature extraction, lightweight SE attention, residual feature reuse, and bidirectional temporal context - contribute more substantially to consistent classification performance across datasets with substantially different characteristics than the availability of additional lead information alone. Collectively, these findings confirm that CaReS-BiNet achieves strong and consistent classification performance across both evaluated datasets, validating its potential as a robust and clinically reliable framework for automated arrhythmia classification across diverse real-world ECG acquisition settings.

### Architectural robustness evaluation on the hybrid single-lead ECG dataset

To further evaluate the architectural robustness of CaReS-BiNet beyond MIT-BIH and PTBDB, the model was independently trained and evaluated on the Hybrid Single-Lead ECG dataset^46^. This experiment was included to further confirm that the proposed architecture is capable of learning relevant and discriminative representations across datasets with substantially different signal characteristics, class structures, and acquisition conditions. The dataset contained 115,693 ECG heartbeat segments across four classes, each represented by 160 amplitude values. A stratified 80:10:10 split was used to preserve class proportions across the training, validation, and test sets, resulting in 92,553 training samples, 11,570 validation samples, and 11,570 independent test samples.

The proposed CaReS-BiNet model achieved an overall test accuracy of 98.08% on the Hybrid Single-Lead ECG dataset. The precision, recall, and F1-score were 98.09%, 98.08%, and 98.08%, respectively, demonstrating strong overall classification performance across the four-class setting. The complete class-wise performance is summarized in Table 4.

**Table 4:** Classification performance of CaReS-BiNet on the hybrid single-lead ECG test set.

Class	Precision (%)	Recall (%)	F1-score (%)	Support	AUC
Class 0	99.84	99.74	99.79	5,785	1.0000
Class 1	90.56	88.02	89.27	643	0.9972
Class 2	98.82	97.89	98.35	3,413	0.9992
Class 3	93.61	96.65	95.11	1,729	0.9978
Average	98.09	98.08	98.08	11,570	–
Overall accuracy	–	–	98.08	11,570	–

**Fig. 9:**
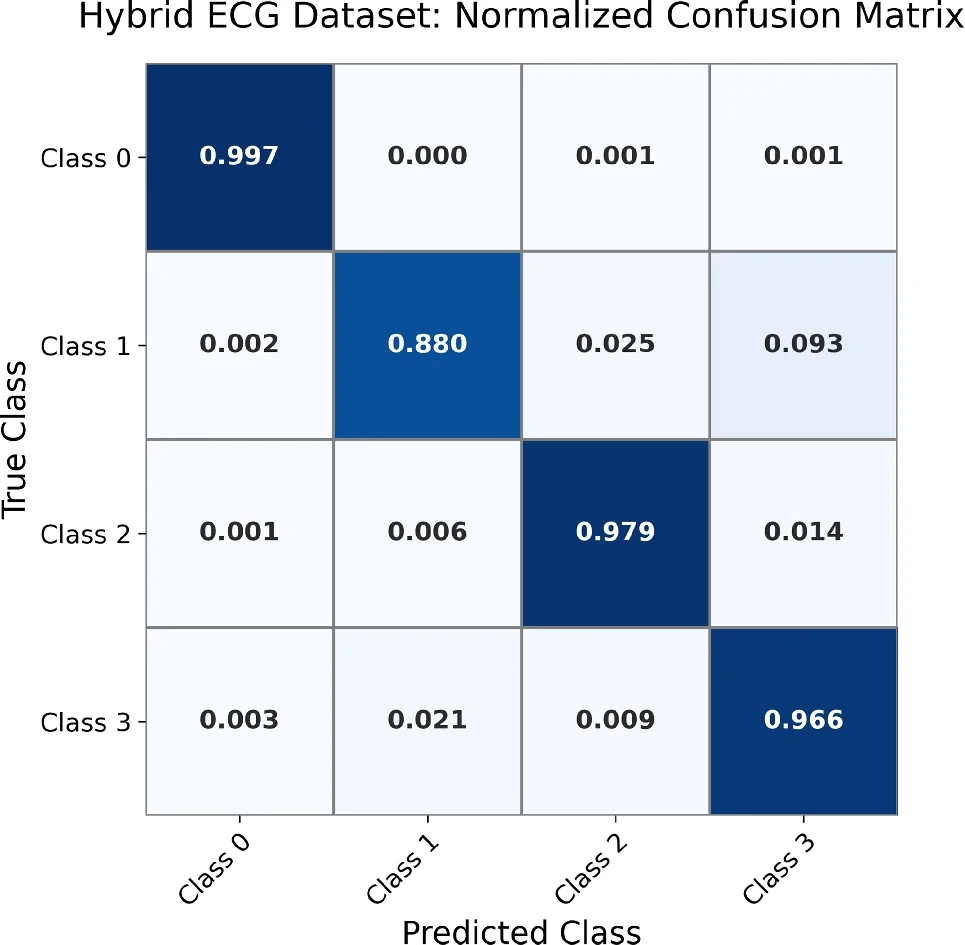
Normalized confusion matrix for the hybrid single-lead ECG test set.

The normalized confusion matrix shown in Fig. 9, further supports the reliability of the proposed model across the four-class task. Class 0 showed near-perfect recognition, with a recall of 99.74%, while Class 2 and Class 3 achieved recalls of 97.89% and 96.65%, respectively. Class 1 was the most challenging class, with a recall of 88.02%. The main source of error for Class 1 was misclassification as Class 3, accounting for 9.33% of Class 1 test samples. Similarly, a smaller proportion of Class 3 samples was misclassified as Class 1 (2.08%). These results indicate that most errors were concentrated between Class 1 and Class 3, while confusion involving Class 0 and Class 2 remained minimal. The strong diagonal dominance of the confusion matrix demonstrates that CaReS-BiNet learned discriminative representations across the hybrid ECG classes rather than overfitting to a single dominant class.

**Fig. 10:**
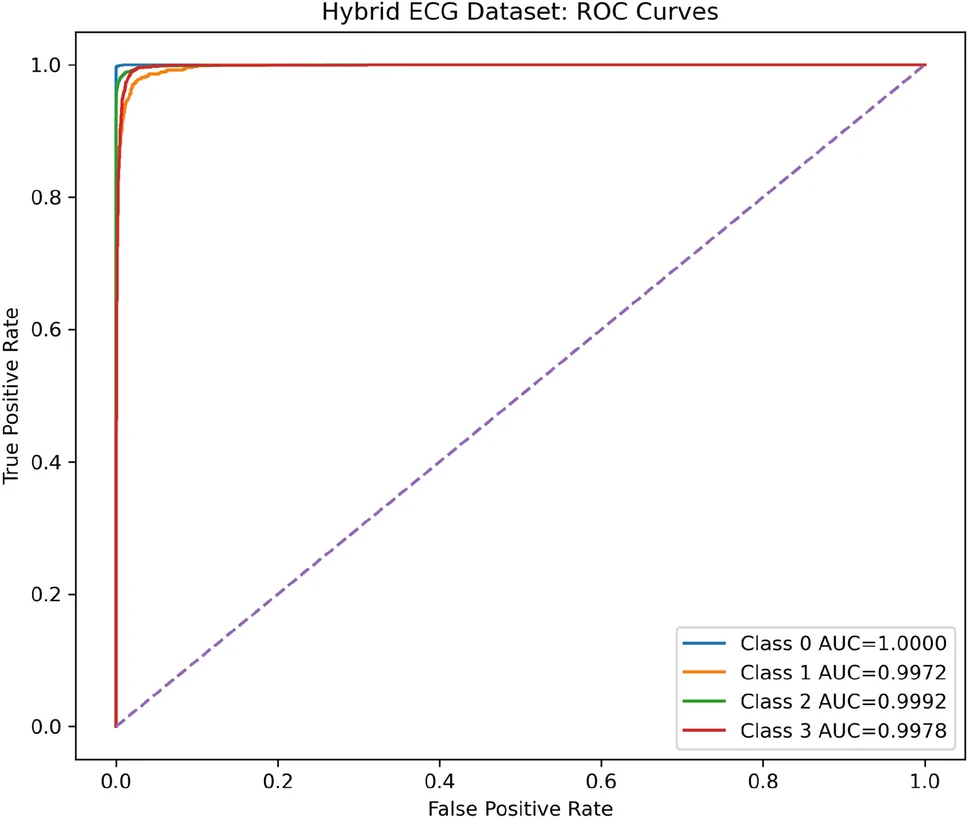
ROC curves for the hybrid single-lead ECG test set.

Further, the ROC analysis in Fig. 10 showed excellent threshold-independent discriminative performance across all classes. The AUC values were 1.0000 for Class 0, 0.9972 for Class 1, 0.9992 for Class 2, and 0.9978 for Class 3. The ROC curves remained close to the top-left corner for all four classes, confirming that the model produced highly separable probabilistic predictions even for the lower-support classes. This result is particularly important because it demonstrates that the comparatively lower recall of Class 1 was not due to poor class separability overall, but rather to a narrower decision boundary between Class 1 and Class 3 at the selected operating threshold.

**Fig. 11:**
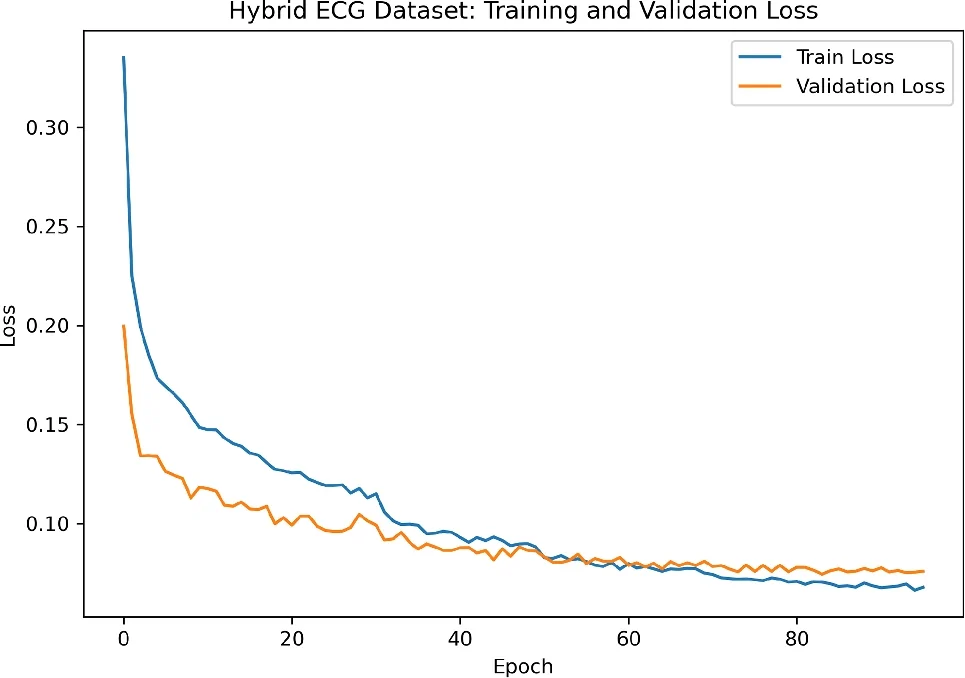
Train and validation loss curves of hybrid single-lead ECG dataset.

**Fig. 12:**
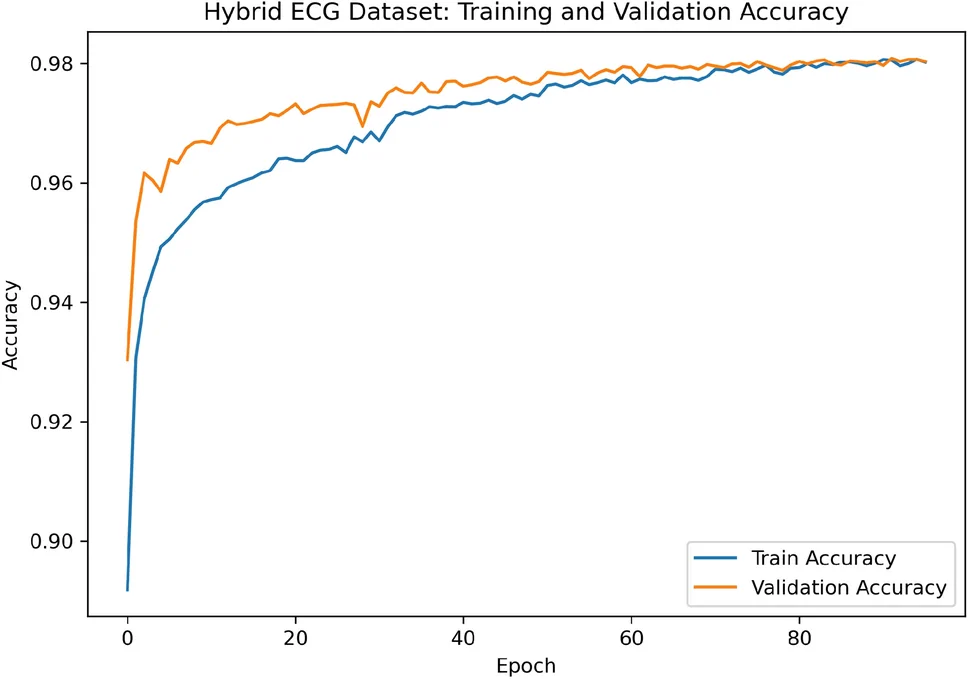
Training and validation accuracy curves of hybrid single-lead ECG dataset.

To confirm that the model is not overfitting, training and validation accuracy and loss curves of CaReS-BiNet on the Hybrid Single-Lead ECG dataset are analyzed. The training and validation accuracy and loss curves are shown in Figs. 12 and 11, respectively, demonstrating stable convergence throughout training. Validation accuracy increased rapidly during the early epochs and then gradually stabilized close to the training accuracy, reaching approximately 98% by the later epochs. The loss curves showed a consistent decrease in both training and validation loss, without marked divergence between the two trajectories. This indicates that the model did not exhibit overfitting despite the larger and heterogeneous nature of the hybrid dataset. The stable learning behavior further supports the effectiveness of the regularization strategies used in CaReS-BiNet, including dropout, L2 regularization, batch normalization, and training-time Gaussian noise injection.

**Table 5:** Ablation study results on MIT-BIH and PTBDB datasets.

Experiment/Dataset	Model architecture	Accuracy (%)
MIT-BIH	CNN only	97.20
	CNN + BiLSTM	98.36
	CNN + Residual + BiLSTM	98.59
	CNN + Residual + SE + BiLSTM	98.74
PTBDB	CNN only	98.25
	CNN + BiLSTM	99.04
	CNN + Residual + BiLSTM	99.18
	CNN + Residual + SE + BiLSTM	99.31
Data Augmentation (MIT-BIH)	CNN + Residual + SE + BiLSTM (without augmentation)	98.16
	CNN + Residual + SE + BiLSTM (with augmentation)	98.74

### Ablation study

Table 5 presents the results of a systematic component-wise ablation study conducted on both the MIT-BIH and PTBDB datasets to evaluate the individual contribution of each architectural component to the overall classification performance. The baseline CNN-only model achieves an accuracy of 97.20% on MIT-BIH and 98.25% on PTBDB, establishing the discriminative capacity of the convolutional feature extraction stage alone. While these results are competitive, the purely convolutional architecture is limited to learning local morphological patterns within individual beat windows and cannot capture the inter-beat temporal dependencies that are diagnostically informative for rhythm-based arrhythmia classes.

The addition of the BiLSTM layer improves accuracy to 98.36% on MIT-BIH and 99.04% on PTBDB, representing gains of 1.16 and 0.79 percentage points respectively. This improvement confirms that temporal sequence modelling across the beat provides complementary discriminative information beyond what morphological features alone can offer. The BiLSTM processes the convolutional feature sequences in both forward and backward directions, enabling the model to encode rhythmic context, such as compensatory pauses following premature ventricular contractions and irregular inter-beat intervals characteristic of supraventricular ectopic beats, that a purely convolutional architecture inherently cannot capture.

The subsequent addition of residual connections further improves accuracy to 98.59% on MIT-BIH and 99.18% on PTBDB, gains of 0.23 and 0.14 percentage points over the CNN+BiLSTM configuration. Residual connections facilitate effective gradient flow through the deeper layers of the network by providing shortcut pathways that prevent vanishing gradients during backpropagation, enabling the model to learn finer morphological distinctions between arrhythmia classes without performance degradation from increased network depth. The consistent improvement across both datasets confirms that residual learning contributes meaningfully to classification accuracy independent of dataset characteristics.

The full proposed architecture, incorporating the SE attention mechanism alongside CNN, residual connections, and BiLSTM, achieves the highest accuracy of 98.74% on MIT-BIH and 99.31% on PTBDB, representing improvements of 0.15 and 0.13 percentage points over the CNN+Residual+BiLSTM configuration. The SE attention mechanism adaptively recalibrates channel-wise feature responses by learning to emphasise the most discriminative feature channels for each arrhythmia class while suppressing less informative ones. Although the incremental gain from SE attention is smaller in magnitude compared to the BiLSTM and residual contributions, it consistently improves performance across both datasets, confirming that channel-wise attention provides a complementary and additive benefit to the temporal and structural components of the architecture. Collectively, the ablation results demonstrate that each architectural component contributes a measurable and consistent improvement, validating the design rationale of the proposed CNN-Residual-SE-BiLSTM architecture.

Furthermore, the contribution of Gaussian noise-based data augmentation is evaluated by comparing the full proposed architecture trained with and without augmentation on the MIT-BIH dataset. As shown in Table 5, training without augmentation yields an accuracy of 98.16%, while incorporating Gaussian noise injection during training improves accuracy to 98.74%, a gain of 0.6 percentage points. This improvement, while modest in absolute terms, is clinically meaningful in the context of arrhythmia classification where marginal gains in accuracy translate directly to improved detection of rare but life-threatening arrhythmia classes. The augmentation strategy injects zero-mean Gaussian noise with a standard deviation of *σ = 0.01* into the training signals, simulating realistic perturbations arising from sensor noise, motion artifacts, and acquisition variability while preserving the underlying physiological morphology of ECG waveforms. By increasing intra-class signal diversity without altering class labels or waveform structure, the augmentation discourages the model from memorising dataset-specific amplitude templates and instead promotes the learning of robust, morphology-based discriminative features. This generalization benefit is particularly important given the class imbalance inherent to the MIT-BIH dataset, where minority arrhythmia classes such as S and F are substantially underrepresented. Augmentation effectively increases the sample diversity of these classes, reducing the model’s bias toward the dominant normal beat class and contributing to the improved balanced classification performance observed in the final model.

### Computational complexity and inference efficiency

To address the clinical applicability of the proposed CaReS-BiNet model, computational complexity was evaluated in terms of the number of trainable parameters, floating-point operations (FLOPs), and inference latency. The analysis was performed under inference mode, where Gaussian noise and dropout layers are inactive. FLOPs were estimated analytically at the layer level using the convention that one multiply-add operation corresponds to two FLOPs. Inference latency was measured after warm-up runs on the same trained model using TensorFlow 2.18.0 on a Linux workstation equipped with an NVIDIA GeForce RTX 4070 GPU and 28 CPU cores.

**Table 6:** Computational complexity and inference efficiency of CaReS-BiNet.

Dataset	Input shape	#classes	Test samples	#Parameters	Trainable parameters	Non-trainable parameters	FLOPs /beat	MFLOPs/ beat	GMACs/ beat	Single-beat latency (ms)	Batch-32 latency/ beat (ms)	Batch-32 throughput (beats/s)
MIT-BIH	187 x 1	5	21,892	1,534,989	1,534,477	512	9,272,813	9.273	0.00464	31.478	0.986	1014.68
PTBDB	187 x 1	2	2,911	1,534,602	1,534,090	512	9,272,042	9.272	0.00464	31.497	0.994	1005.77
Hybrid Single- Lead ECG	160 *×* 1	4	11,570	1,321,868	1,321,356	512	7,929,964	7.930	0.00396	29.655	1.092	915.41

As shown in Table 6, the proposed CaReS-BiNet model required approximately 1.53 million parameters for both datasets, with only a small difference between MIT-BIH and PTBDB due to the final softmax classification layer having five output classes for MIT-BIH and two output classes for PTBDB. The estimated computational cost was approximately 9.27 MFLOPs per ECG beat segment for both datasets, corresponding to 0.00464 GMACs per beat. This indicates that the added residual, squeeze-and-excitation, and BiLSTM components do not introduce excessive computational burden.

In terms of inference speed, the median single-beat inference latency was approximately 31.48 ms for MIT-BIH and 31.50 ms for PTBDB. Under batch-wise inference with a batch size of 32, the per-beat latency decreased substantially to 0.986 ms/beat for MIT-BIH and 0.994 ms/beat for PTBDB, corresponding to throughputs of 1014.68 beats/s and 1005.77 beats/s, respectively. These results show that CaReS-BiNet supports rapid beat-level ECG inference while maintaining a moderate parameter count and low per-segment computational cost. The test-set inference further confirmed the practical efficiency of the proposed model. CaReS-BiNet processed the MIT-BIH test set of 21,892 beats in 0.684 s, corresponding to 0.031 ms/beat and 31,997.08 beats/s. For PTBDB, the model processed 2,911 test samples in 0.135 s, corresponding to 0.046 ms/beat and 21,521.64 beats/s. For the Hybrid Single-Lead ECG dataset, the model processed 11,570 test samples in 1.276 s, corresponding to 0.110 ms/beat and 9066.15 beats/s. These results demonstrate that CaReS-BiNet supports rapid ECG beat-level inference across all evaluated datasets. Therefore, the proposed framework is computationally feasible for offline ECG screening and near real-time clinical decision-support workflows, particularly when batch-wise inference is used.

## Conclusion

This paper proposed CaReS-BiNet, a Convolutional and Residual Squeeze-and-Excitation BiLSTM-based Network for automated ECG arrhythmia classification, integrating multi-scale convolutional feature extraction, residual learning, lightweight Squeeze-and-Excitation channel attention, and BiLSTM temporal modelling into a unified end-to-end framework. The proposed architecture was designed to address four core limitations of existing methods, dependence on handcrafted features, insufficient temporal dependency modelling, limited evaluation beyond single controlled benchmarks, and the persistent challenge of class imbalance in arrhythmia classification, through principled architectural choices supported by Gaussian noise augmentation for class imbalance mitigation and validated through a systematic ablation study. Evaluated on the MIT-BIH Arrhythmia Database under the AAMI EC57 five-class protocol, CaReS-BiNet achieved an accuracy of 98.74%, precision of 98.70%, recall of 98.73%, and F1-score of 98.71%, outperforming the majority of compared state-of-the-art methods on recall and F1-score. Binary VEB and SVEB detection further confirmed clinical reliability, with SVEB precision of 97.99% and recall of 94.64% substantially exceeding existing methods. Architectural robustness was further validated through independent evaluation on the PTB Diagnostic ECG Database and the Hybrid Single-Lead ECG dataset, demonstrating strong and consistent classification performance across datasets with substantially different acquisition characteristics, class structures, and classification tasks. These findings collectively demonstrate that the proposed architecture is capable of learning relevant and discriminative representations across heterogeneous datasets, providing strong evidence that principled architectural design contributes more substantially to consistent performance than dataset-specific tuning or additional lead information. The ablation study further confirmed that each component contributes measurably and independently, with BiLSTM providing the largest gain, validating the rationale of the proposed hierarchical design. In addition, the computational complexity analysis showed that CaReS-BiNet requires only approximately 1.53 million parameters and 9.27 MFLOPs per ECG beat, with batch-wise inference throughput exceeding 1000 beats/s, indicating that the model is computationally feasible for practical ECG screening applications.

Despite these contributions, the inherent morphological similarity between minority classes S and F and normal beats presents a persistent challenge in ECG arrhythmia classification, a difficulty well-documented across the literature regardless of architectural choice. Nevertheless, CaReS-BiNet demonstrates competitive minority class performance relative to existing state-of-the-art methods, confirming the effectiveness of the proposed hierarchical architecture and augmentation strategy in partially mitigating this challenge. Future work will explore extending CaReS-BiNet to multi-lead ECG input to exploit richer spatial cardiac information to further improve minority class sensitivity.

## Data Availability

The datasets used and/or analysed during the current study are available from the corresponding author on reasonable request.

## Footnotes

1 https://www.who.int/news-room/fact-sheets/detail/cardiovascular-diseases-(cvds)

2 https://www.who.int/health-topics/cardiovascular-diseases

3 https://physionet.org/content/ptbdb/1.0.0/
